# Self-healing of damage inside metals triggered by electropulsing stimuli

**DOI:** 10.1038/s41598-017-06635-9

**Published:** 2017-08-02

**Authors:** Hui Song, Zhong-jin Wang, Xiao-dong He, Jie Duan

**Affiliations:** 1 School of Astronautic, Harbin Institute of Technology, Harbin, China; 2 The national key laboratory for precision hot forming of metals, Harbin Institute of Technology, Harbin, China; 3 School of Materials Science and Engineering, Harbin Institute of Technology, Harbin, China

## Abstract

The microscopic defects that distributed randomly in metals are not only hard to detect, but also may inevitably cause catastrophic failure. Thus, autonomic probing and healing for damage inside metals continue to be a challenging. Here we show a novel approach for self-healing using electropulsing as a stimulus to trigger repairing of damaged metals. This is achieved via a process that through expelling absolutely currents, the microcrack causes them to be redistributed to form a concentrated and a diluted region around it, thereby inducing an extremely high temperature gradient and a large compressive stress, which drive material flow to close microcracks. Simultaneously, a large enough heat for bonding atoms was produced. That is, the microcrack as an empty cavity can be regarded as a special micro-device to shape a localized microscopic energy field, which in turn activates a healing process. The microstructure and mechanical property verified the extrinsic self-healing of a titanium alloy. The process is performed on a short timescale, is enable to detect automatically and act directly on the internal defects in metals, and to heal damage without any healing agent, long time heating as well as applied high pressure, offering unique advantages over conventional healing approaches.

## Introduction

Materials that unavoidably affected by various external effects such as mechanical, thermal, radiation and so on, may subject to damage or even failure, which reduces safety and service life cycle of structures. Therefore, it is highly desirable to heal or repair damage in materials via an internal or external stimulus. Biological systems are known to have the intrinsic self-healing ability^1^, namely, the damage itself as a stimulus triggers them to generate and/or deliver and release healing agents to the defect sites, achieving autonomous healing of injury^1, 2^. Self-healing in metals^1, 3^, however, is intrinsically difficult^3–5^ because they do not have the ability to probe and identify damage, and their atomic mobility or diffusivity is so low and melting points are so high that a healing process is difficult to activate^6^, consequently introducing energy and matter into the metallic materials become a prerequisite for triggering necessary interactions as well as healing.

A self-healing approach for metallic materials is one that based on microcapsules embedded in matrix, via which healing agents was introduced into alloys, and released after mechanical damage^1, 3, 7^. Despite the effective restoration of electrical conductivity of metals^8^, the interfacial bonding between the healing agent and the matrix is poor^3^, lowering the mechanical properties of the materials. Another way is the so-called precipitation healing^1, 3, 9, 10^ where the precipitation phase serves as an intrinsic healing agent. With an external stimulus such as heat, solute precipitates that more likely occur at high-stress/high-energy regions, will autonomously seek and localize at the site of damage, achieving the self-healing of metals. But application of this approach is limited to those precipitating alloys. Analogously, by high temperatures diffusion of atoms, microdefects in alpha-Fe can be healed^11^. Yet the diffusion path in metals is very small, leading to healing of defects with a rather small volume (nanoscale), and high temperatures may lead to degradation of the material.

In the absence of extrinsic healing agents and intrinsic precipitation phases, traditionally, a large hot plastic deformation was introduced into a bulk metal under the combined processes of high temperature and high pressure^12, 13^, leading materials to flow for closing cavities and/or microcracks. This method overcomes limitations of atomic mobility or diffusivity, and also bypasses the issues of detecting defect sites, whereas accompanied by significant energy consumption and degradation of the mechanical properties.

Damage is a localized structural defect induced by deformation or radiation etc. To avoid degradation of the original matrix, it should be beneficial to *in-situ* entirely eliminate or heal these defects. So ideal healing approaches should be ones that are enable to detect automatically and act directly on the internal defects in metals. These tasks are particularly challenging in the case of microscopic defects because the damage is distributed randomly in metals and often hard to detect^2^. Despite precipitation effect meets these requirements, it does not have a general applicability for most metals and alloys. Therefore, it is very attractive to find a universal triggering effect promoting that repairing was localized in the damage region, and then the extrinsic self-healing can be achieved in metals and alloys.

Although without the ability to restore automatically their properties like biological materials do, metallic materials can respond to external stimuli. For instance, the composite materials containing magnetic nanoparticles sense actively to an external magnetic field, the magnetic filler can be used to trigger healing by locally heating the composite under an external alternating magnetic field^14–17^. Analogy to the magnetic field, the electric field is sensitive to the conductivity of metals^18–24^. And the damaged metals, from the perspective of electrodynamics, are also considered to be a special composite material composed of metallic matrix and empty cavities (microcracks). In other words, while microcracks inside metals are generally known as the defect and the damage, they also can be regarded as a special micro-device that absolutely expels the current^20^, causing current crowding and local Joule heating^24^, via which a highly localized energy field may be formed around the microcrack. This implies that the electric current could autonomously sense and respond to the site of damage.

Obviously, this localized energy field that act directly on the microcracks can be controlled via adjusting pulsed current density and period, thus the conditions for healing microcracks in solids may be met. Therefore, electropulsing can be used as a stimulus to trigger self-healing of damaged metals, and has a general applicability for most metals and alloys. To test this hypothesis, by multi-physics environments coupling, we simulate the spatial distribution of electric current, local energy landscape, temperature, temperature gradient and Von mises stress around the microcrack, and verify the possibility of self-healing microcracks in metallic materials via electropulsing by the experiments of mechanical properties and the microstructure observation.

## Results and Discussion

### The spatial distributions of electric current and localized energy landscape around the microcrack

It is hard to measure the temperature and monitor the experimental phenomenon inside metals. In this paper, in order to estimate the temperature, the temperature gradient and the stress induced by electropulsing, using ANSYS Multiphysics/LS-DYNA software platform to simulates the coupled multi-physics environments (A detailed description of finite element (FE) model are given in Supplementary).

Based on numerical simulations, firstly, we take the electric current, the temperature, the temperature gradient and the Misses stress at 26 μs as an example to illustrate the electric current redistribution and the energy landscape at microcrack regions.

**Figure 1:**
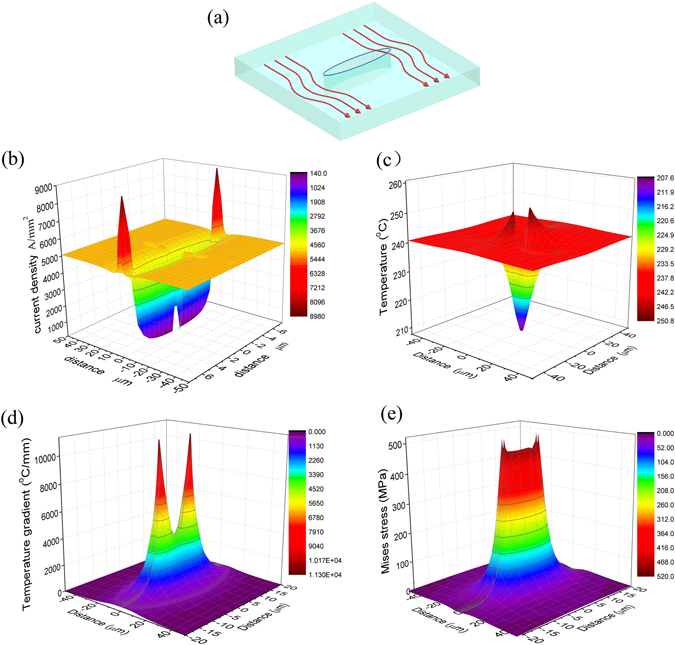
Electric current redistribution and localized energy landscape induced by the microcrack at t = 26 μs (**a**) Schematic of electric current distribution. (**b**–**e**) the three-dimensional representation of the current density, temperature, temperature gradient and Mises stress on a cut plane across the microcrack, respectively.

The microcrack inside metals can be regarded as a cavity with infinite resistance that does not allow a current to pass^20^ (Fig. 1(a)), so the conductivity inside damaged materials is not uniform. The local current density distribution in the materials with the inhomogeneous electrical conductivity is governed by the microscopic Ohm’s law:*j*_*i*_ = *σ*_*iK*_ *E*_*K*_^24^, *σ*_*iK*_ is the conductivity tensor field, *E*_*K*_ is the local electric field. The configuration of an electric current around the microcrack in damaged materials will be changed due to microscopic discontinuity of conductivity. For a more vivid display this feature of localized heterogeneous distribution, Fig. 1(b) shows the three-dimensional representation of the electric current density distribution on a cut plane across the microcrack. It can be found that the microcrack expels the current into the matrix, i.e. the electric current was redistributed in titanium alloys due to the microcrack. The electric current detours the microcrack, causing high current density at the microcrack tip, forming a concentrated region of the current, and thus the current density around the microcrack tip is the highest, reached up to 8000~9000 A/mm^2^ that was greater than 5090 A/mm^2^ of the original matrix. There are two sharp peaks in the three-dimensional view of the current density distribution, which correspond to the microcrack tip (Fig. 1(b)).

It can be seen from Fig. 1(b) that the current density in both sides of the microcrack decreased sharply, the density value decreased from 5090 A/mm^2^ to 140 A/mm^2^ within the range of several micrometers, that is, the current density is “diluted” (forming a diluted region), which correspond to a mountain valley in three-dimensional representation of the current density distribution on a cut plane across the microcrack. It implies that the microcrack force current redistribute in different region of metals, a heterogeneous current density distribution is produced around the microcrack. The current lines are not disturbed in the region far away from the microcrack, showing that the current detect automatically and target at the microcrack.

If electrical energy is dissipated in metals, they will be converting into heat, according to Joule’s law:

1 $$Q={I}^{2}Rt=\frac{{j}^{2}tV}{\sigma }$$

where, *Q*-the energy, *I*- the current, *R*-the resistance, *j*-the current density, *t*- time, *σ*- the conductivity,*V*-the volume. According to eq. (1), the larger the current density is, and the larger the heat energy is.

The temperature rise Δ*T* by the Joule heating:

2 $${\rm{\Delta }}T=\frac{Q}{cm}=\frac{{j}^{2}t}{\sigma c\rho }$$

where, *c*- the specific heat, *ρ*- the density, so it is can be found from eqs (1) and (2) that although the conductivity of the matrix is homogeneous, temperature around the microcrack is not uniform if current density distribution is heterogeneous.

**Figure 2:**
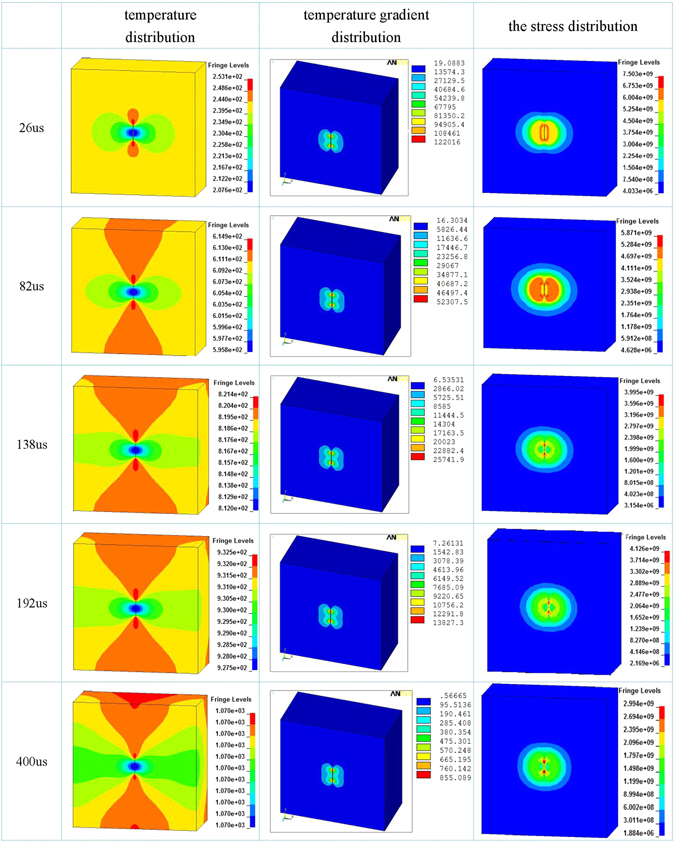
The time evolution of localized temperature field, temperature gradient and Von mises stress (the unit of the quantities in the Figure is system of electromagnetic units).

At t = 26 μs, the temperature in the microcrack tip is 253.1 °C, the temperature in matrix without microcracks is 239.5 °C, the temperature difference between them achieve 13.6 °C in the range of several micrometers (Figs 1(c) and 2). The temperature distribution in both sides of the microcrack (in the diluted region) is divided into several areas, the temperature in the regions adjacent the microcrack sides is 207.6 °C, the temperature increased up to 216.7 °C~230.4 °C in the range of a distance that is about several micrometers away from the microcrack sides. Owing to nonuniformity of the temperature rise, the temperature gradient will be produced around the microcrack

3 $$gradsT=\frac{\partial T}{\partial l}$$

where, *l* is the distance. It is can be found from eq. (3) that the temperature gradient is inversely proportional to the distance *l*. Despite the temperature difference is only 10~20 °C, but there exists a large temperature gradient within the range of several micrometers around the microcrack, the magnitude of the temperature gradient reached up to about 4,000 °C/mm, value of the temperature gradient at the microcrack tip is more great (reaches as high as 10,000 °C/mm), which is more considerable than that of the matrix region, i.e., the temperature gradient around the microcrack change abruptly (Fig. 1(d)).

Inhomogeneous temperature rise will induce thermal compressive stress in materials. The temperature gradient can be regarded as a measure of inhomogeneity of the temperature rise. In the other word, such lager temperature gradient is concentrated and highly localized around the microcrack, which will produce high compressive stress at the microcrack region. It is found from Fig. 1(e) that the stress in both sides of the microcrack (in the diluted region) reached up to about 600 MPa, and the stress at the microcrack tip is as high as 750 MPa. This stress field is also highly localized. Therefore, the pulse current is not only enabling to detect automatically the site of damage, but also induce the compressive stresses that act directly on the internal defects in metals.

### The time evolution of temperature, temperature gradient and Von mises stress field

To understand the healing process of damage, the evolution of temperature, temperature gradient and Von mises stress field with times was also revealed by FE simulations (Fig. 2). The temperature increases with time due to the increasing external current and Joule heating effect, and when time increases to 82 μs, the temperature in the matrix reached up to 609.2 °C, but the temperature in the microcrack area is still not uniform, the temperature at the microcrack tip is 613~614.9 °C, the temperature in both sides of the microcrack is 595.8~607.3 °C (Fig. 2), and high and low temperature region was spread compare with t = 26 μs, showing thermal diffusion in the titanium alloy. The value of the temperature gradient in both sides of the microcrack reached up to about 2000 °C/mm, the one at the microcrack tip is as high as 5230.7 °C/mm, leading to that a compressive stress field of 469.7~528.4 MPa is produced in both sides of the microcrack (Fig. 2), which higher than the yield strength of TC4 titanium alloy at about 600 °C.

When time increases to 138 μs, the temperature in the matrix reached up to 818.6 °C, and high and low temperature region was expanded constantly compare with t = 26 and 82 μs, the temperature difference between the microcrack tip and the matrix, as well as between the microcrack sides and the matrix is also decreased. The value of the temperature gradient in both sides of the microcrack and at the microcrack tip are 858.5 °C/mm and 2574.1 °C/mm, respectively, corresponding compressive stress are 324.9 MPa and 463.9 MPa (Fig. 2).

Similar results are shown when time increases to 192 μs, but the temperature gradient and the compressive stress are gradually decreased with time. At t = 400 μs, the temperature difference between maximum and minimum temperatures is only 0.3 °C, the temperature of total body reached up to 1070 °C that provide the energy conditions for atoms diffusion and microcrack healing (Fig. 2). Metals is good conductor, and the temperature gradient is the driving force of heat diffuse, implying that given sufficient time, the heat in spatially localized high temperature region will conduct to the surrounding. This is why temperature tends to be uniform distribution as t = 400 μs.

The decreasing in the temperature gradient and the compressive stress with time showed that it is necessary to accumulate enough energy around the microcrack in a short period of time so as to produce sufficient high temperature gradient and large compressive stress for closing microcracks, otherwise effect of heat conduction makes the temperature in metal to be uniform, the temperature gradient and the compressive stress will disappear, indicating that whether healing can eventually be achieved is determined by the competition between the localized energy landscape and the heat conduction. To achieve the temperature gradient and the compressive stress, the electric pulse should be tailored to have suitable parameters (current density and period), such that the microcrack was closed and healed before the temperature distribution tends to be uniform.

Of course, there are restrictions on the scale of defects. In previous reports^25–28^, for instance, using the electric current to a plate with the artificial cracks (its magnitude reached up to millimeter level), melting of metal could be caused by the strong detour effect, and a molten hole may be formed in the melting area. In this case, cracks cannot be healed, only is arresting or retarding crack propagation.

### Plastic deformation and microcrack closing

**Figure 3:**
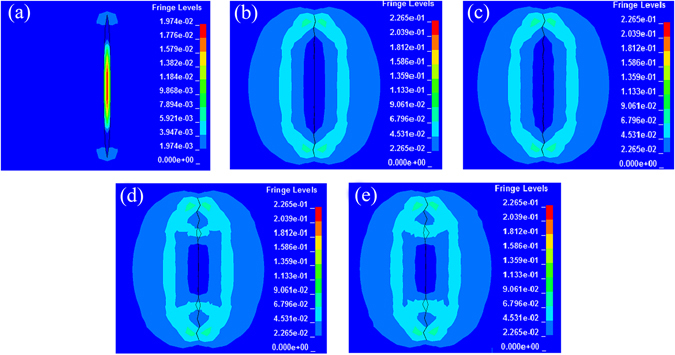
Strain variation and microcrack width versus time (the unit of the quantities in the Figure is system of electromagnetic units) (**a**) 26 us; (**b**) 82 us; (**c**) 138 us; (**d**) 192 us; (**e**) 400 us.

In the absence of both extrinsic and intrinsic healing agents, plastic deformation is used to close the microcrack, which is an important requirement for atomic interactions because microcrack healing can only be achieved within the range of atomic interactions. It can be known from the above results that the heterogeneous current distribution induced a very large temperature gradient and a compressive stress around the microcrack, which is sufficient large to deform the TC4 titanium alloy. It is found from Fig. 3 that at t = 26 μs, plastic deformation occurred only at the microcrack tip, and width of the microcrack is reduced slightly. When time increases to 82 μs (see Fig. 3 and Supplementary Videos S1 and S2), a localized strain region occurred around the microcrack, and the microcrack is closed, showing the TC4 titanium alloy around the microcrack is deformed under the compressive stress (Fig. 3). During subsequent process, strain regions around the microcrack remain, implying that the atoms on the surface of the closing microcrack can interact.

### Microcrack closing and atomic interaction

Healing or repairing of damage is a process that physically separated parts are bonded by chemical or physical method. It is an essential process for healing damage that the atoms on the surface of the microcrack can interact. In the absence of any healing agent, atomic interactions can only be achieved within the range of atomic interactions. This requires that electropulsing should be enabling to close the microcracks while bonding atoms. Hence, a healing should at least include two courses: namely the microcrack closing stage and atomic interactions stage.

**Figure 4:**
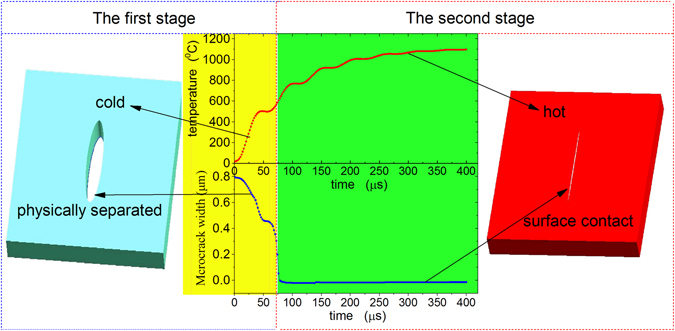
Comparison of temperature rise and microcrack width versus time during the whole electropulsing process.

The comparison of temperature rise and microcrack width versus time during the whole electropulsing process was showed in Fig. 4; it is found that these courses can be divided into two stages. In the first stage, matrix is at low temperature, and the microcrack in a physically separated state is closing gradually. In the second stage, the microcrack surfaces are brought into contact with each other under the compressive stress, the microcrack was closed, and separated surface is in the range of atomic interactions, and at the same time matrix is heated up to high temperatures, which make microcrack healing possible.

The simulation results on healing damaged are also observed on the microcrack with length of 14 μm and the angle between the current direction and the microcrack direction are 0°, 30° and 60° (see Supplementary Fig. S10(a,b)),as well as the microcrack with length of 6 μm (see Supplementary Fig. S10(c)).

### Experimental demonstration of microcrack healing. Observation of morphology of microcracks

**Figure 5:**
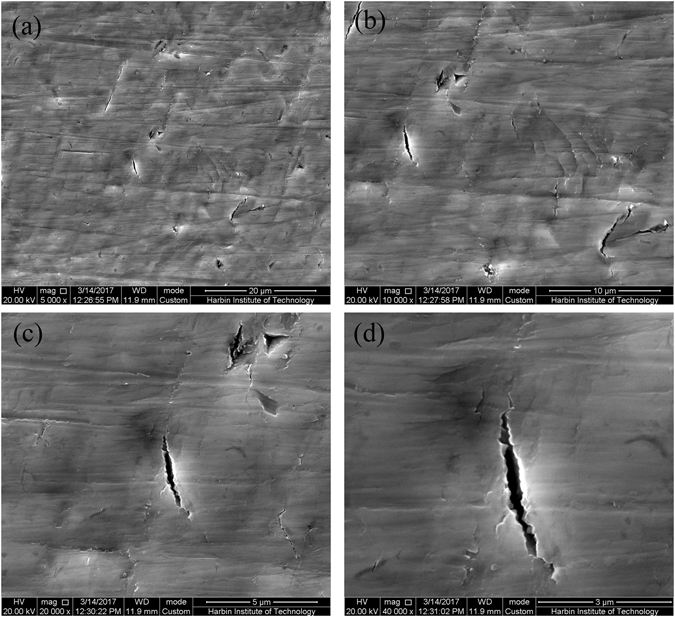
Typical morphology of microcracks of damaged titanium alloy sheet before electropulsing. (**a**–**d**) are Magnified 5,000, 10,000, 20,000 and 40,000 times SEM image, respectively.

In fact, in order to obtain the more practical results, it is very important to select the randomly distributed microcracks in metals as the research object. The plastic deformation is employed to obtain these microcracks in titanium sheet alloy^29^ (see Methods and Supplementary Fig. S2). The morphology of some typical microcracks with different length and direction is shown in Fig. 5 and Supplementary Fig. S15. In order to make these microcracks more clear, SEM images with different magnification are given. We observed also the deformation-induced surface roughening that is frequently found in tension.

**Figure 6:**
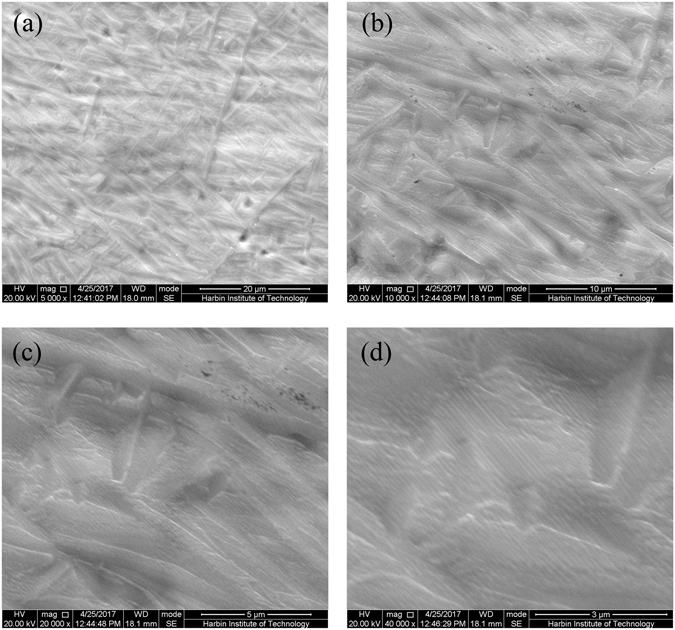
Typical morphology of surface of damaged titanium alloy sheet after electropulsing. (**a**–**d**) are Magnified 5,000, 10,000, 20,000 and 40,000 times SEM image, respectively.

The healing effect appears after electropulsing, and microcracks are completely healed (Fig. 6 and Supplementary Fig. S16), irrespective of their size and direction. No damage could be found on the specimens. It should be mentioned that surface morphology of the TC4 titanium alloy was changed compared with that before electropulsing, fine acicular microstructures occur in the electropulsed TC4 titanium alloy, which may be caused by high heating and cooling rate on the specimen surface^30^. Based on the simulation results, the temperature rise caused by electropulsing in the TC4 titanium alloy was estimated to be 1070 °C, which is higher than the phase transition temperature (980 °C) of the TC4 titanium alloy. Therefore, the phase transition may occur during electropulsing, in which acicular microstructures can be formed due to martensitic transformation^31^. Because surfaces interact with the gas argon at room temperature, the highest cooling rate on the top surface during the cooling course was obtained^30^, which restrict the growth of the grains, thus fine acicular microstructures are observed on the surface of electropulsed TC4 titanium alloy.

**Figure 7:**
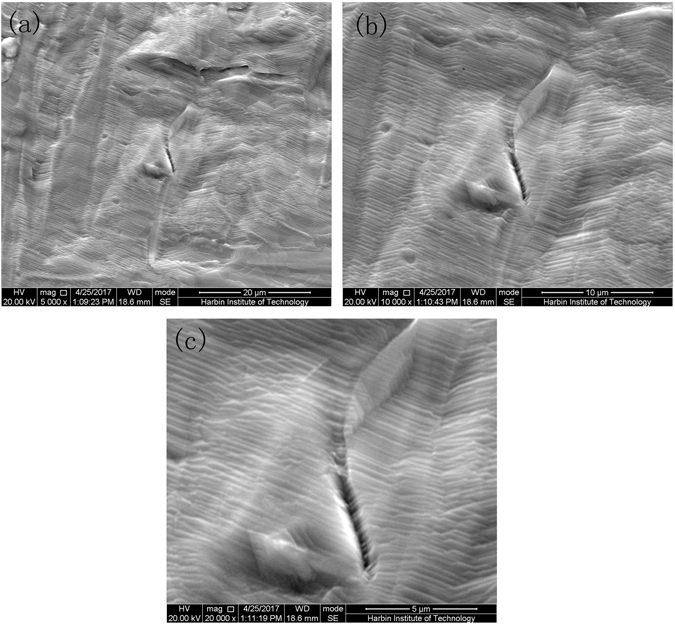
Typical morphology of microcracks of damaged titanium alloy sheet after high temperature treatment. (**a**–**c**) are Magnified 5,000, 10,000 and 20,000 times SEM image, respectively.

In order to further demonstrate the mechanism by which healing damage in the titanium alloy during electropulsing process, a conventional high temperature heating treatment was carried out as a comparison. In this process, longer heating time is used, but there only exists thermal effect which leads to uniform temperature rise in the titanium alloy. The results show that the microcracks are not healed completely by the conventional high temperature heat treatment (Fig. 7 and Supplementary Fig. S13). Some microcracks still exist on specimens after heat treatment (Fig. 7 and Supplementary Fig. S13), and the deformation-induced surface roughening are still remained. The surface morphology of undamaged titanium alloy was also observed (Supplementary Fig. S14). It can be seen that although the grain remarkably grows, no damage was observed. It demonstrates that the residual microcracks (Fig. 7 and Supplementary Fig. S13) are produced in the pre-deformation, not by heating treatment. That is, the conventional heat treatment in which there are no localized temperature gradient and the compressive stress, does not eliminate microcracks produced by the pre-deformation. Furthermore, it should be noted that lamellar structures are also found in the TC4 titanium alloy after the conventional high temperature heating treatment (Supplementary Fig. S13), which implies phase transition occurs also during the heating treatment like the electropulsing process. So phase transition should not be regarded as the reason of the damage was healed.

### Uniaxial tensile mechanical properties

**Figure 8:**
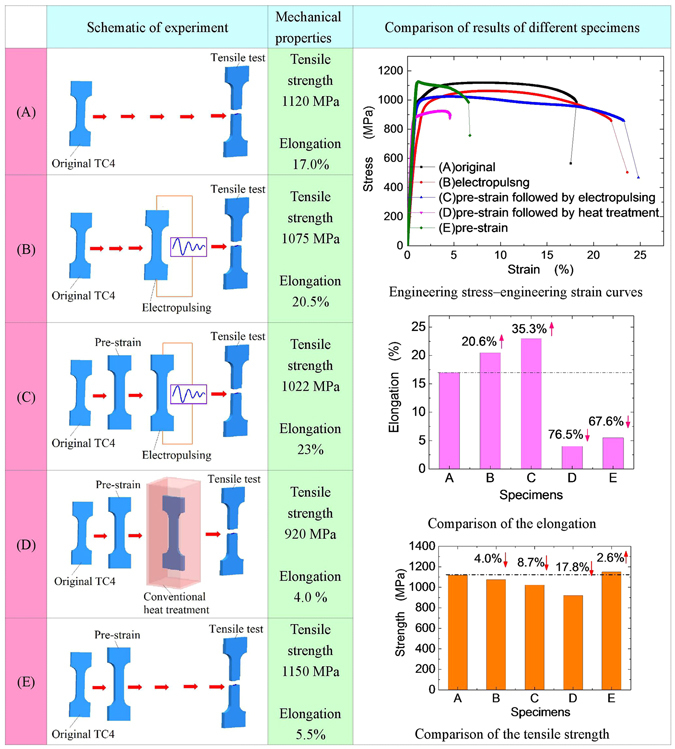
Schematic of the experiment conditions and comparison of the results.

The change of mechanical properties is another sign of producing and healing of damage in metals. To investigate the effect of electropulsing on the damage evolution in titanium alloys, all tensile specimens are divided into five groups (shown in Fig. 8). Specimens A are not subjected to both pre-strain and electropulsing treatment, used as the reference specimen. Specimens B are also not subjected to pre-strain, but are treated by electropulsing so as to compare the influence of electropulsing. For specimens C, D and E, they are first pre-stained with strain of 10% to generate damage by tension and unloaded. And then specimens C and D are treated by electropulsing and conventional heat treatment, respectively. After pre-strained and unloaded, and then specimens E are stretched to failure finally.

The typical engineering stress–strain curves are shown at top right in Fig. 8. The elongation of original TC4 sheet (Specimens A) is 17.0%, the tensile strength is 1120 MPa. By contrast, pre-strain specimens E only has 5.5% elongation owing to the deformation damage, but deformation hardening leads to a slight increase in strength of up to 1150 MPa.

After the electropulsing, the elongation of specimens B increases to 20.5%, increasing by 20.6% compared with the original specimens A, but the tensile strength decreases slightly from 1120 MPa to 1075 MPa (decreasing by 4.0%). For pre-strained specimens C, the electropulsing increases further its elongation up to 23% (increasing by 35.3% relative to specimens A), which means that the damage by pre-strain in titanium alloys was healed completely. The tensile strength is reduced to 1022 MPa, decreasing by 8.7% compared with the original specimens A. Therefore the comprehensive mechanical properties of the titanium alloy healed by electropulsing are not degraded. In addition to damage healing, the electropulsing may cause the change of microstructure. The lamellar structure often leads to increase in the strength, which is in contradiction with the result observed for electropulsed TC4 alloys. This shows that the phase composition may also change, for example the high-temperature martensitic phase β-Ti (is more soft than α-Ti) remained in a Ti alloy^31^, from which decreasing in the tensile strength of electropulsed titanium alloy is believed to originate. Detailed discussion of this problem is beyond the scope of this paper.

In stark contrast, after heat treatment, the tensile strength of pre-stained TC4 (specimens D) decreases to 920 MPa, the elongation decreases to 4.0%, which means that the damage is not healed and the mechanical properties are degraded by long time heating.

Comparisons of the elongation and the tensile strength for different specimens are shown at right middle and bottom right of Fig. 8. The results of mechanical properties are consistent with the observation of morphology of microcracks, showing electropulsing enable to trigger self-healing of damaged metals.

In summary, the pulse current autonomously responds to the site of damage and was redistributed to form a concentrated region and a diluted region at the tip and sides of the microcrack respectively. As a results of the heterogeneous distribution, high current density as well as short duration, the extremely high temperature gradients and the large compressive stress are produced around the microcrack, which firstly deforming the TC4 titanium alloy to close microcracks, and then the microcrack surfaces are brought into contact with each other under the compressive stress. Subsequently, the matrix is heated up to high temperature, providing the energy for atomic interactions, thus damage was healed successfully.

This targeted healing of damage has following striking characteristics, the electropulsing can be conducted in open air in the absence of any conventional heating source and/or applied high pressure, and healing agents also not required. Electropulsing offer unique advantages over conventional healing approach, and is a cleaner, more efficient and green method, and has a general applicability for most metals and alloys.

## Methods

The experimental material in this study is an annealed TC4 titanium alloy sheet, and its thickness is 1.0 mm. The tensile dog-bone specimens are cut from TC4 sheet, and the size of specimen’s parallel portion is 15 mm in length, and 6 mm in width.

For producing the randomly distributed microcracks in TC4 sheet, specimens are stretched on a test machine to produce pre-strain first, and then unloaded. To investigate the damage evolution of sheet metal, specimens are polished before stretching so as to observe the morphology of microcracks on the surface of the deformed specimens using scanning electron microscopy (SEM) (see Supplementary Fig. S2).

The tensile tests at room temperature are conducted on an Instron 5569 test machine equipped with extensometer with gauge 10 mm to examine mechanical properties, the tensile test speed is 1.0 mm/min.

The damaged specimens are treated by electropulsing using the capacitor banks discharge. The waveform of electropulsing is a damped oscillationwave, the values of period are same (t_p_ = 110 μs), but the values of maximum current density are different (see Supplementary Fig. S9). The electropulsing time is very short (about 400 μs). After electropulsing treatment, the morphology of microcracks of polished specimens is also observed by SEM.

Conventional high temperature heating treatment of damaged titanium alloy is carried out in a vacuum furnace at 1150 °C for 0.5 h (see Supplementary Fig. S12).

## Electronic supplementary material

Supplementary Information

Supplementary Video S1

Supplementary Video S2
