# Supplementary material for: Self-healing of damage inside metals triggered by electropulsing stimuli
Source: Sci Rep. 2017 Aug 2;7:7097. doi: 10.1038/s41598-017-06635-9 (PMC5540974; doi:10.1038/s41598-017-06635-9)
Supplement: Supplementary file 1 — Supplementary Information [file 41598_2017_6635_MOESM1_ESM.doc]

Supplementary Information for

**Self-healing of damage inside metals** **triggered by electropulsing stimuli**

Hui Song^1^, Zhong-jin Wang^2, 3^*, Xiao-dong He^1^, Jie Duan^3^

1. School of Astronautic, Harbin Institute of Technology, Harbin, China;

2. The national key laboratory for precision hot forming of metals, Harbin Institute of Technology, Harbin, China; 3. School of Materials Science and Engineering, Harbin Institute of Technology, Harbin, China

Correspondence and requests for materials should be addressed to Z.-J.Wang. (e-mail: [wangzj@hit.edu.cn](mailto:wangzj@hit.edu.cn))

**1. Finite element modeling**

**1.1 Method of stereology distribution of particles derived from the section distribution.**

Nucleation, growth and coalescence of small internal microcracks are the dominant damage mechanism in metals at ambient temperature. The size and density of microcracks is associated with plastic deformation [1, 2 and 3]. An important problem that we need to face is determining how large is the size of microcracks when damage occurs at material? How much is their volume density? Quantitative observations on microcracks nucleation and growth as a function of strain during tensile tests are presented [1, 2 and 3]. Nevertheless, the most quantitative models on the number of microcracks per unit area as function of strain are built based on observation using 2-D sections. Volume density of microcracks has not yet been addressed quantitatively.

The distribution function of crystal sizes on a cross-sectional surface can be obtained by direct observation. These models have been developed and enhanced over the past 40 years, allowing predictions of volume fraction of crystal sizes [4, 5 and 6]. Given a microdefect of radius R in a body with unit volume (Fig.S1.), then the probability of a 2D profile section the microdefect is 2R/1=2R.


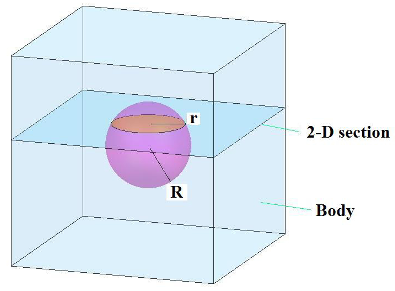


Supplementary Figure S1 the cross-sectional surface of a spherical particle on a 2-D section

A relationship between the distributions function $F(r)$and the volume distribution function $f(R)$is [4]:

$F(r)dr=2\int_{R}^{\infty} \frac{2r}{\sqrt{R^{2}-r^{2}}}f(R)dR\cdot dr$ (s1)

$F(r)=2\int_{R}^{\infty} \frac{rf(R)dR}{\sqrt{R^{2}-r^{2}}}$ (s2)

$f(R)=-\frac{1}{\pi}\int_{R}^{\infty} \frac{Rdr}{\sqrt{R^{2}-r^{2}}}\frac{d}{dr}\left(\frac{F(r)}{r}\right)$ (s3)

$f(R)=\frac{R}{\pi}\int_{R}^{\infty} \frac{F(r)dr}{r^{2}\sqrt{R^{2}-r^{2}}}-\frac{R}{\pi}\int_{R}^{\infty} \frac{1}{r^{2}\sqrt{R^{2}-r^{2}}}\frac{dF(r)}{dr}dr$ (s4)

If$f(R)=\delta(R-a)$, after discretization of continuous variable, relationship between the distributions function on sections $F(r)$and the volume distribution function $f(R)$can be expressed as:

$f(n\delta)=\frac{1}{\pi}\sum_{m} \left\{-\int_{\theta_{m,n}}^{\theta_{m+1,n}} \frac{dF(r)}{dr}d\theta+\frac{1}{n\delta}\int_{\theta_{m,n}}^{\theta_{m+1,n}} F(r)d\sin\theta\right\}$ (s5)

Where,$r=R\sec\theta$,$(n+m)\delta=n\delta\sec\theta_{m,n}$,$\cos\theta_{m,n}=\frac{n}{n+m}$.

$f_{n}=2\delta\cdot f(n\delta)=\frac{1}{\pi\delta}\sum_{m=0} \left\{\frac{1}{2n}\left(F_{n+m}+F_{n+m+1}\right)\int_{\theta_{m,n}}^{\theta_{m+1,n}} \cos\theta d\theta-\frac{1}{n\delta}\left(F_{n+m+1}-F_{n+m}\right)\left(\theta_{m+1,n}-\theta_{m,n}\right)\right\}$ (s6)

$f_{n}=\frac{1}{\pi\delta}\sum_{m=0} A_{n,m}F_{m+n}$ (s7)

Where,$A_{n,0}=\left(\frac{1}{2n}\sqrt{1-\left(\frac{n}{n+1}\right)^{2}}+\cos^{-1}\frac{n}{n+1}\right)$

$$A_{n,m}=\frac{1}{2n}\left(\sqrt{1-\left(\frac{n}{n+m+1}\right)^{2}}+\sqrt{1-\left(\frac{n}{n+m-1}\right)^{2}}\right)+\cos^{-1}\frac{n}{n+m+1}+\cos^{-1}\frac{n}{n+m-1}-2+\cos^{-1}\frac{n}{n+m}$$

**1.2 Estimation of microcracks size and their volume density by combining theory with SEM observation**

Inspired by predicting model of volume fraction of crystal sizes, we consider the microcracks inside metals as a special grain (Fig.S2), and then average density and size of internal microcrack**s** can be determined by equation (s7). The morphology of microcracks on the surface of the deformed specimens is observed using scanning electron microscopy (SEM) (Fig.S2), and the average number of microcracks per unit area and their size are obtained (Fig.S3). According to equation (s7) and SEM experimental results, the size and the volume density of microcracks can be derived (Fig.S4). For TC4 titanium alloy, microcrack scales range from 2μm to 16 μm (Fig.S3 and S4), in which the two typical microcracks with length of 6 μm and 14 μm were selected as research objects.


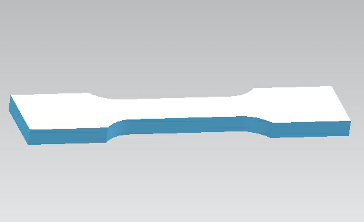

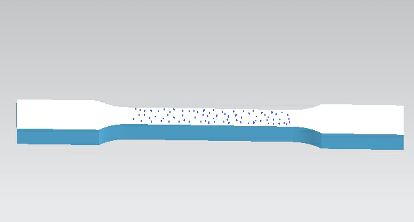

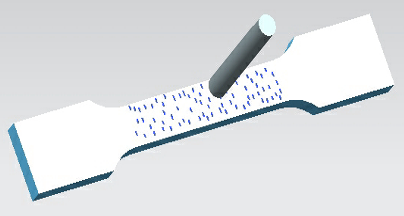


Polishing specimen surface

Producing microcracks by deformation

Observing microcracks by SEM

Determining volume density of microcracks

Units’ body contained a microcrack


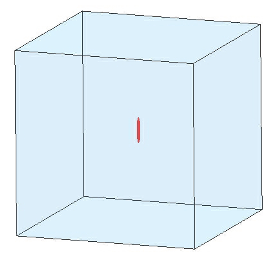

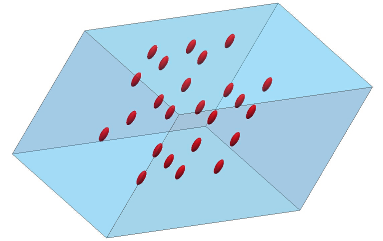

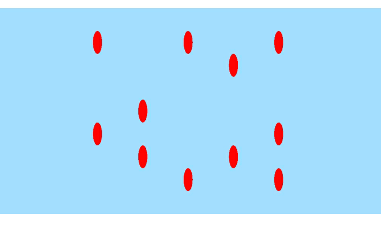


Determining microcracks size and density

Supplementary Figure S2 Schematic diagram of determining density and size of microcracks

The average number of the microcrack with length of 14 μm per unit area is about 27/mm^2^. Combined with equation (s7), volume density of microcracks with length of 14 μm is about 924 /mm^3^, i.e. 1/1082251μm^3^, implying a cube with edge length of 102.7 μm contains a microcrack with length of 14 μm. The average number of the microcrack with length of 6 μm per unit area is about 120/mm^2^. Combined with equation (s7), volume density of microcracks is about 7600 /mm^3^, i.e. 1/131000μm^3^ that corresponding to a cube with edge length of 51μm contained a microcrack with length of 6 μm.


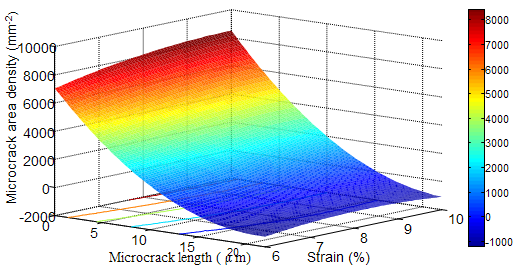


Supplementary Figure S3 relationship among microcracks size and area density and strain


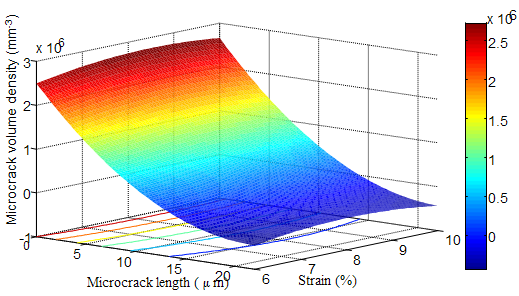


Supplementary Figure S4 relationship among microcracks size and volume density and strain

**1.3 A “loose coupling” method for the coupled multi-physics environments**

When high density electropulsing is applied to metals, the significant Joule heating concentration and the compressive stresses will be occurred around the microcracks. The strain due to the compressive stress may affect the electric current distribution and Joule heating effect at the crack tip. In addition, as the temperature increased, the strength of materials decreased, plastic deformation will occur in titanium alloys under the compressive stresses. Therefore, electropulsing healing of microcracks relates not only to electro-thermo-structural coupling, but also to plastic deformation of materials which is a necessary condition for closing microcracks.

In this paper, systematic electro-thermo-structural coupled-field analyses for the electropulsing healing of microcracks will be solved by ANSYS Multiphysics. Considering the complex non-linear characteristics of the plastic deformation of materials, the explicit code LS-DYNA is used to calculate the whole plastic deformation process. A “loose coupling” method based on the basis of ANSYS Multiphysics/LS-DYNA software platform, is chosen to simulate the coupled multi-physics environments, in which the deformed geometry and temperature loadings are extracted and again applied by user-defined subroutines using APDL language (Fig.S5).

**1.4 Finite element model**

The electric current density field has the r^-1/2^ singularity at the crack tip, which is analogous to stress concentration or singularity in solid mechanics [7]. Usually, the surfaces of the crack are uneven, and show a complicated shape. In order to avoid too high current density singularity at the microcrack tip and simplify the computation, the microcrack is modeled as an elliptical shape (Fig.S6). The original matrix is modeled as a cube with side edge of 100 μm, and the size of microcrack is 14 μm in length, 10 μm in thickness and 0.8 μm in width (Fig.S6).

**ANSYS/LS-DYNA**

Deformation analysis

**ANSYS Multiphysics**

Electro-thermo-structural coupled analysis

**ANSYS Multiphysics/LS-DYNA**

Establishing

the coupled multi-physics environments

Electric current, temperature and stress analysis

Post-processing

Nodal temperature-time history and

Coordinates of nodal configurations

Nodal configuration-time history

Environment initialization

Output nodal load data

Establishing deformation analysis

physics environments

Defining nodal temperature loads

Creating keyword file

Deformation analysis

Post-processing

Supplementary Figure S5 The flow chart for coupled analyses

**
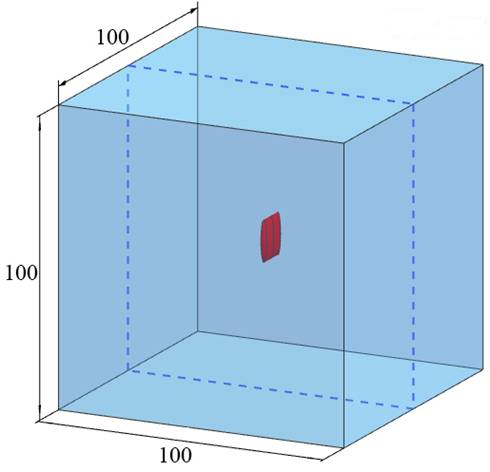
**
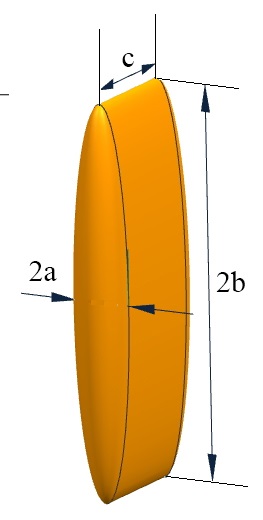


Supplementary Figure S6 the dimension the microcrack and corresponding Units’ body contained it


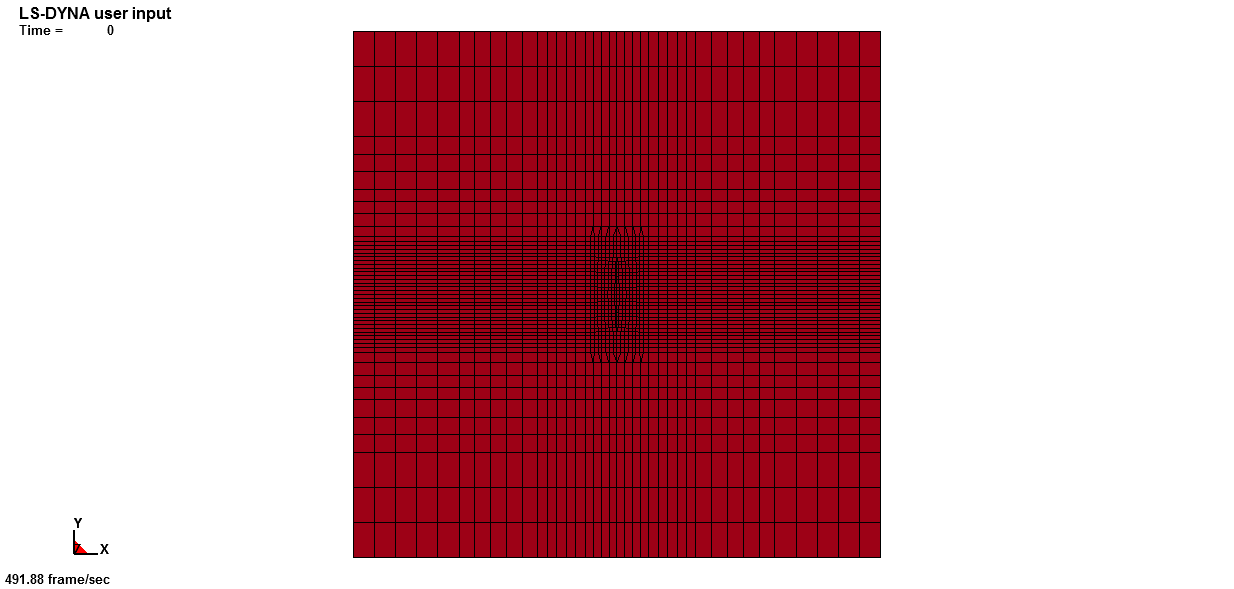

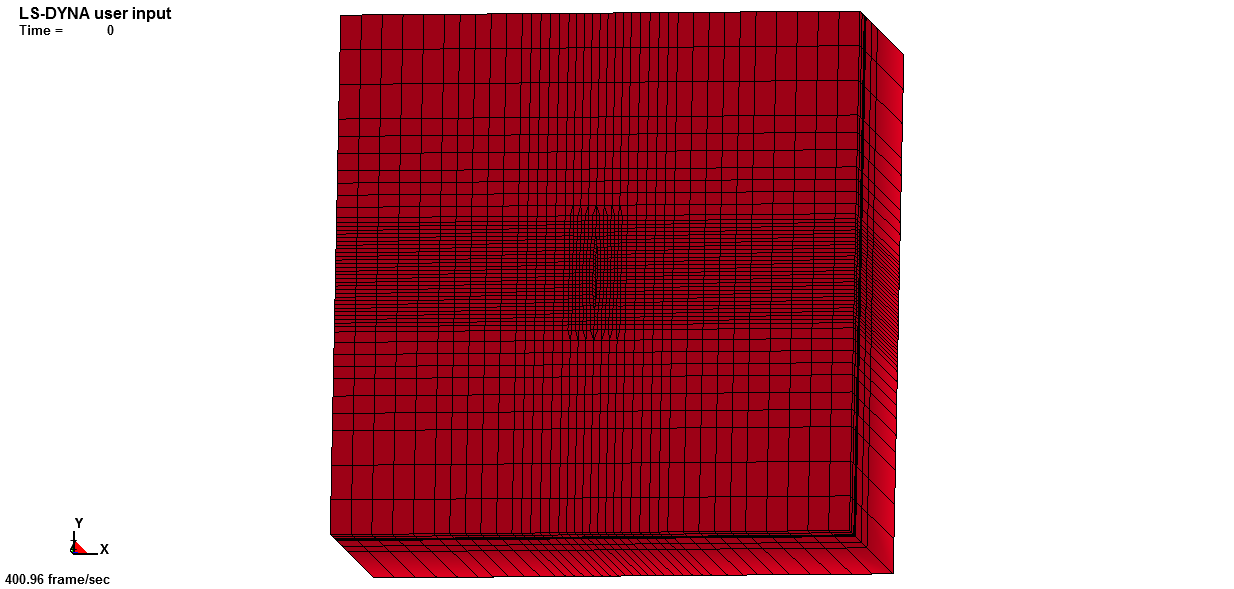


Supplementary Figure S7 Finite element mesh. (a) 2D view; (b) 3D view

The SOLID 5 element type, i.e. the 8- nodes hexahedron element with the electro thermo–-structural coupled field analysis is used in ANSYS Multiphysics. The SOLID164, i.e. the 8-node element with the deformation analysis, are applied to analysis plastic deformation of titanium alloys by ANSYS/LS-DYNA. Due to the symmetry of the problem, only a half of the cube with a microcrack is modeled (Fig.S7).

**1.5 Material parameters and loading conditions**

The plastic stress and strain are analyzed based the von Mises yield criterions [7]. The stress–strain relationships of TC4 titanium alloy at various temperatures is showed in Fig.S8. Temperature-dependent properties of TC4 titanium alloy are presented in Table S1. The phase change effects of the heating process can be ignored to reduce computation time. The pulsed electric current applied in the finite element model is a damped oscillation wave (Fig.S9), and is approximately expressed by:

$I=I_{m}e^{-\alpha t}\sin\omega t=I_{m}e^{-\alpha t}\sin\frac{2\pi}{t_{p}}t=I_{m}e^{-5198t}\sin57100t$ （S8）

Where, the pulse period t_p_ =110 μs, α is the damping exponent, I_m_ is maximum current amplitude.

Supplementary Figure S8. the stress–strain properties of TC4 titanium alloy at various temperatures

Supplementary Figure S9 Waveform of electropulsing

Supplementary Table S1. Temperature-dependent properties of titanium alloy

| Temperature/℃ | Specific heat  J/(kg·℃) | Thermal conductivity  w/(m·℃) | density kg/m^3^ | Coefficient of thermal expansion  10^-6^/℃ | Poisson’s ratio | Resistivit/  μΩ·m | Yong 's modulus  /GPa |
| --- | --- | --- | --- | --- | --- | --- | --- |
| 20 | 610.1 | 6.9 | 4440 | 8.4 | 0.34 | 1.70 | 109 |
| 100 | 623.4 | 7.4 | 4390 | 9.1 | 0.34 | 1.76 | 97 |
| 200 | 653.2 | 8.7 | 4360 | 9.2 | 0.34 | 1.82 | 94 |
| 300 | 675.7 | 9.8 | 4310 | 9.3 | 0.35 | 1.86 | 91 |
| 400 | 690.7 | 10.3 | 4290 | 9.5 | 0.37 | 1.89 | 80 |
| 500 | 702.8 | 11.9 | 4260 | 9.7 | 0.37 | 1.91 | 75 |
| 600 | 712.3 | 13.1 | 4240 | 10 | 0.39 | 1.92 | 66 |
| 700 | 715.6 | 14.1 | 4220 | 10.2 | 0.39 | 1.92 | 48 |
| 800 | 719.8 | 15.2 | 4200 | 10.4 | 0.39 | 1.92 | 35 |
| 900 | 721.3 | 16.3 | 4180 | 10.9 | 0.39 | 1.92 | 30 |
| 1000 | 724.9 | 17.4 | 4160 | 10.9 | 0.39 | 1.92 | 22 |
| 1100 | 726.5 | 18.3 | 4140 | 11 | 0.39 | 1.92 | 15 |
| 1200 | 729.4 | 19.5 | 4120 | 11 | 0.39 | 1.92 | 5 |
| 1300 | 731.8 | 20.6 | 4100 | 11 | 0.39 | 1.92 | 0.1 |

**2. Simulation results under other conditions**

The simulation results on healing damage are also observed on the microcrack with length of 14 μm and the angle between the current direction and the microcrack direction are 0º, 30º and 60º (Fig.S10 (a) and (b)),as well as the microcrack with length of 6 μm (Fig.S10 (c)).

| (a) | 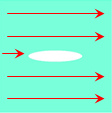 | 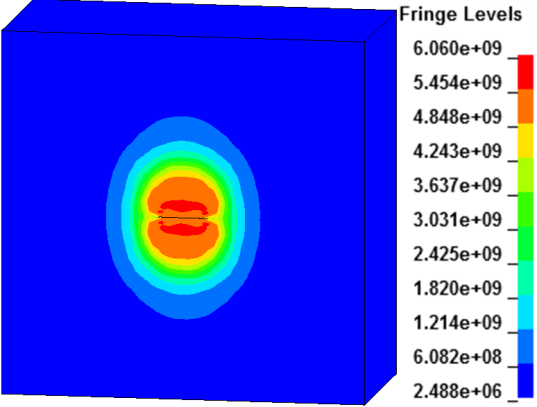 |
| --- | --- | --- |
| (b) | 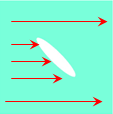 | 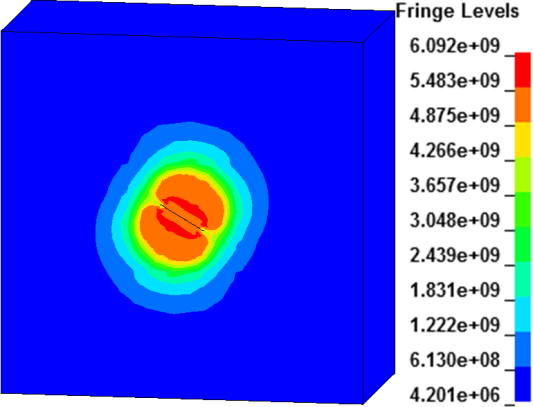 |
| (c) | 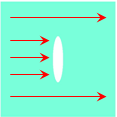 | 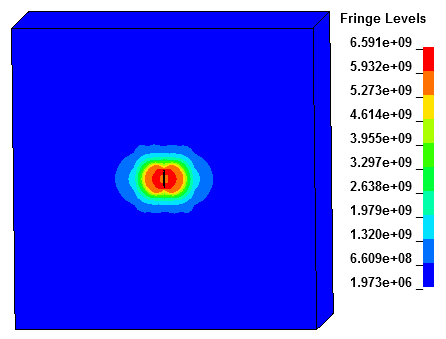 |

Supplementary Figure S10. Simulation results of microcrack healing under different conditions

**3. Electropulsing treatment**

In order to protect the titanium alloy from oxidizing during electropulsing, and make sure that SEM observation not to be affected, placing the polished specimens that connected in series with an electric current source via copper clamps, into a chamber filled of high purity Ar (Fig.S11).

**
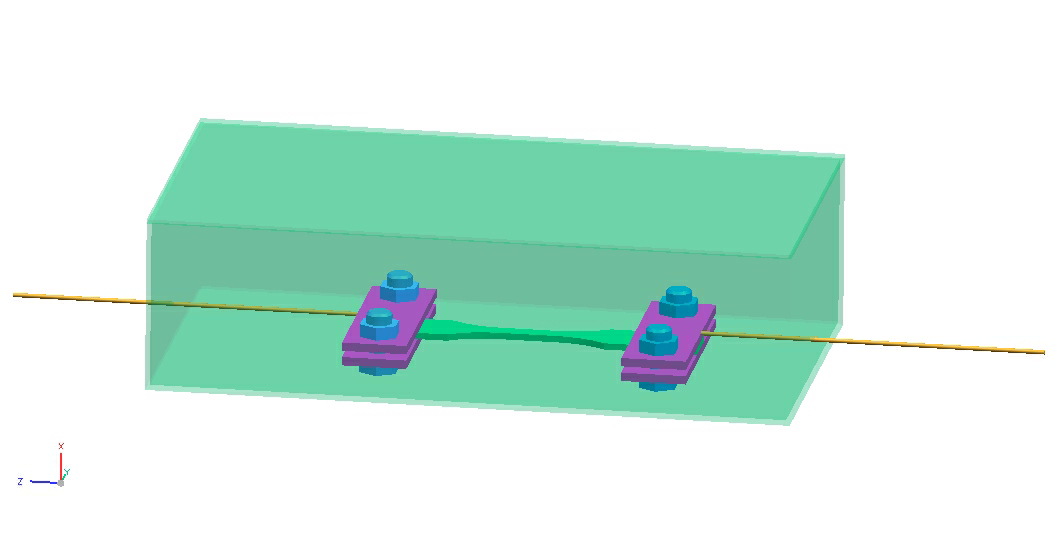
**

Supplementary Figure S11 Schematic diagram of electopulsing in a chamber filled of high purity Ar

**4. Conventional** **high temperature heating treatment**

The specimens for conventional high temperature heating was also polished and damaged by uniaxial tension. The heating treatment was carried out in a vacuum furnace. The specimens was heated to 1150℃ at the vacuum of 1×10^-3^Pa within 2 hour, holding at 1150℃with the vacuum of 9.1×10^-4^Pa~5.3×10^-4^Pa for half an hour that is greater than the electropulsing time of 400 μs, and then cooled with the vacuum of 6.7×10^-5^Pa~5.3×10^-4^Pa, from 1150℃ to 850 ℃ within 1 hour, from 850℃ to 500 ℃within 3 hour, from 500℃ to 200 ℃within 6hour. And the specimens finally cooled to room temperature in the furnace (Fig.S12).

Supplementary Figure S12 process of general high temperature heating treatment


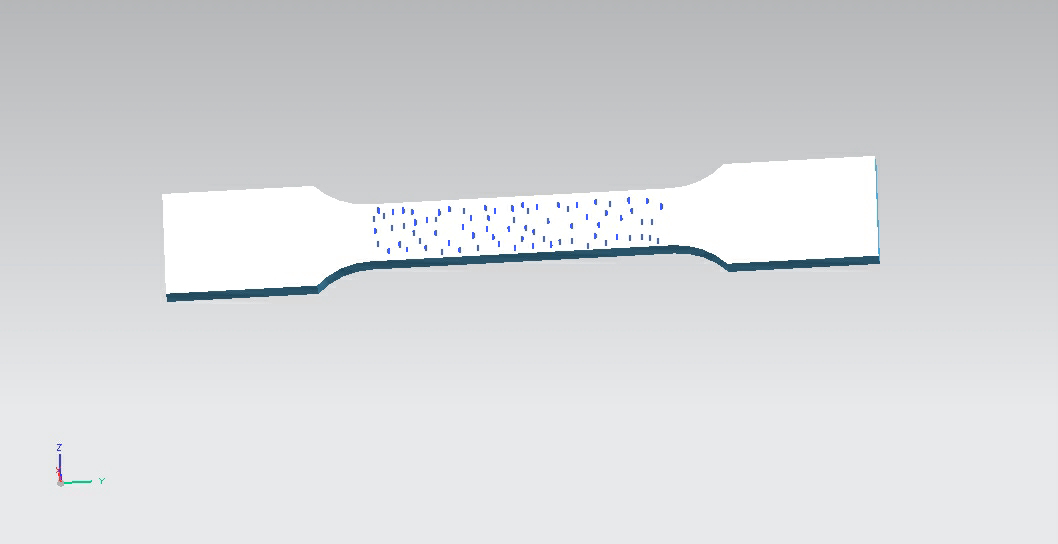


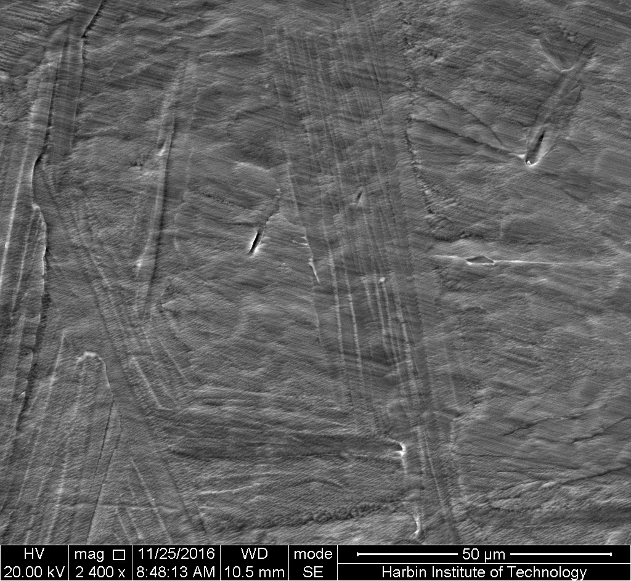

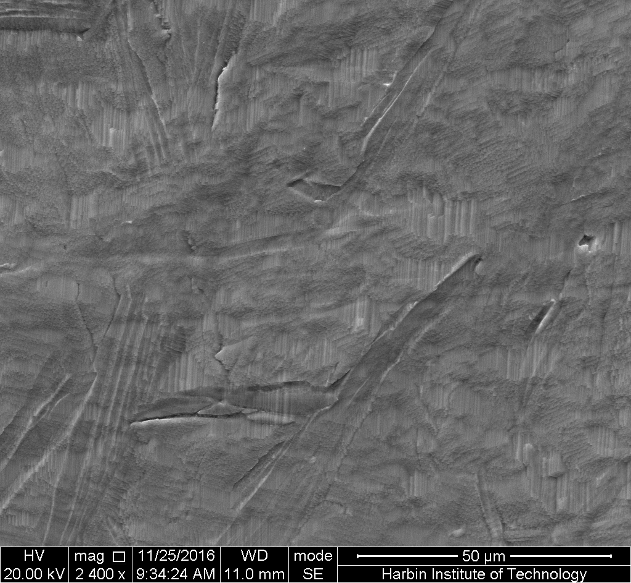


Supplementary Figure S13 typical surface morphology of damaged titanium alloy after heating treatment


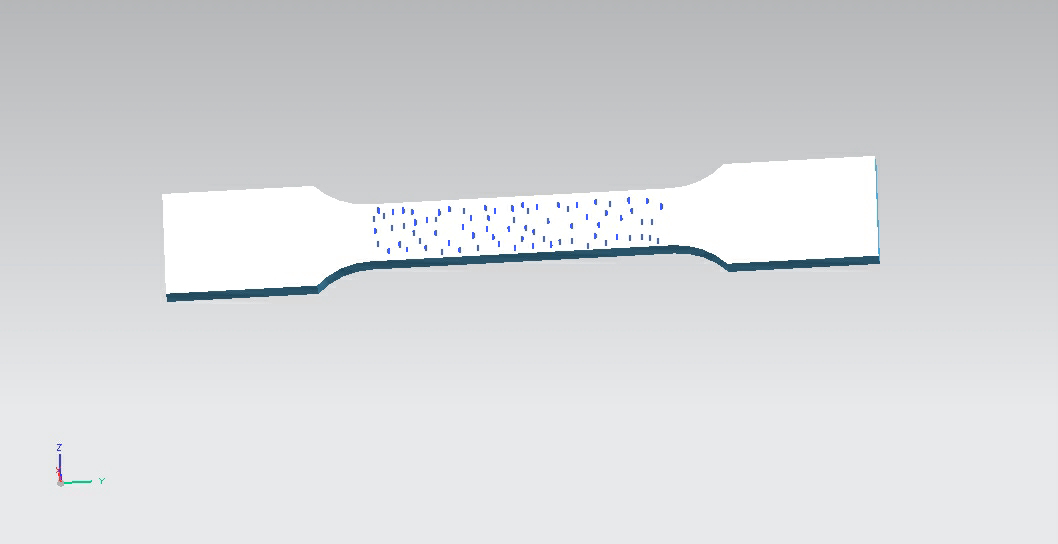


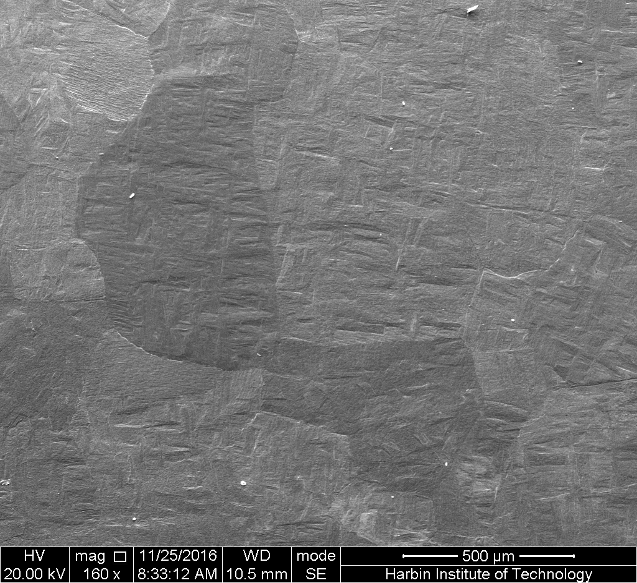

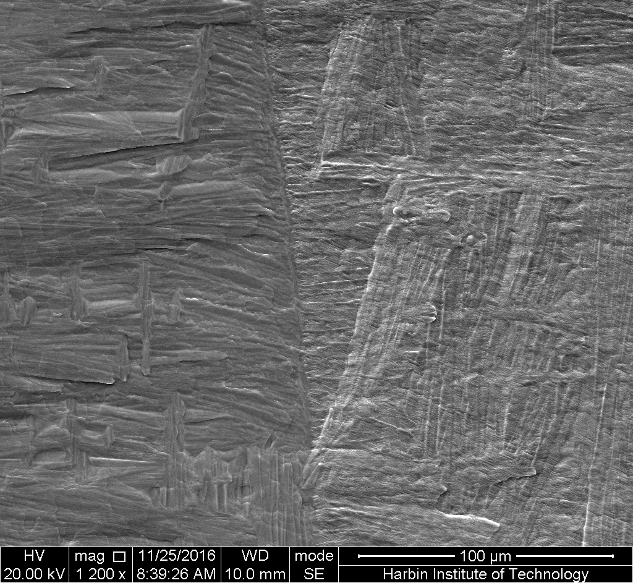


Supplementary Figure S14 typical grain and surface morphology of undamaged titanium alloy after general high temperature heating treatment

**5. Typical morphology of damaged titanium alloy before and after electropulsing**


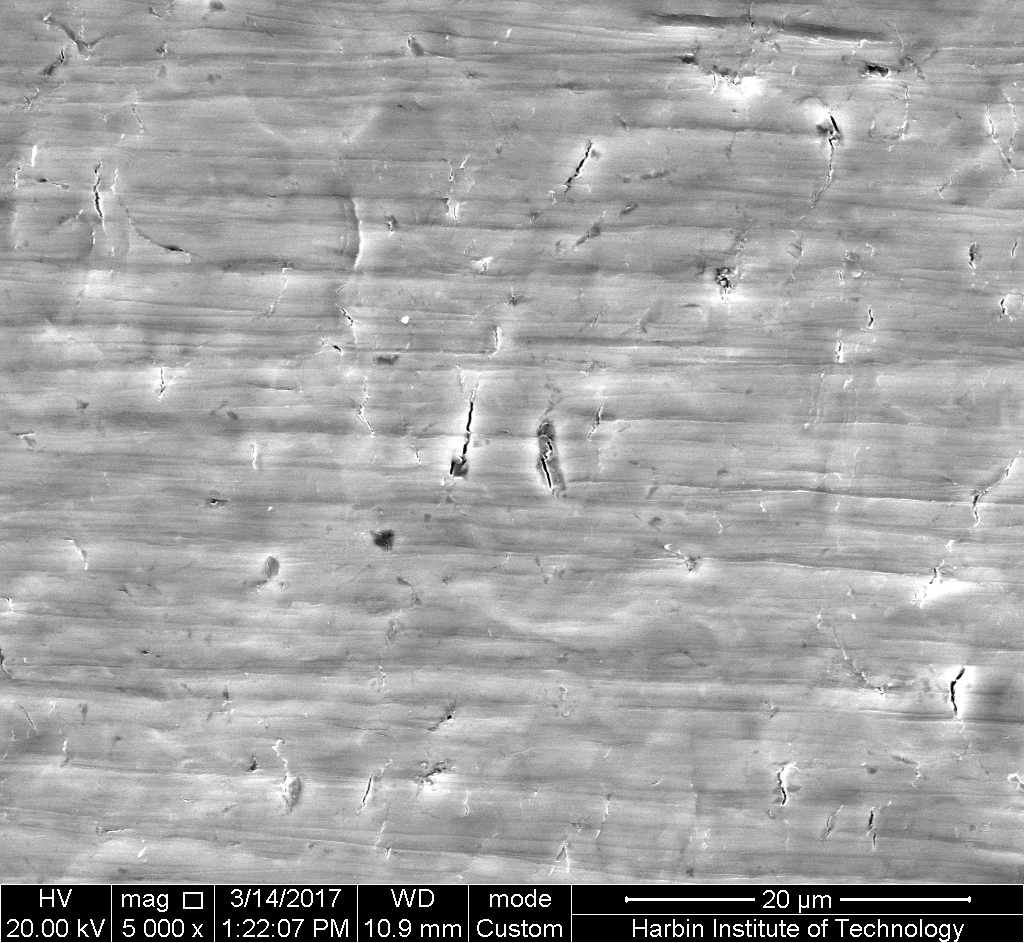


(a)


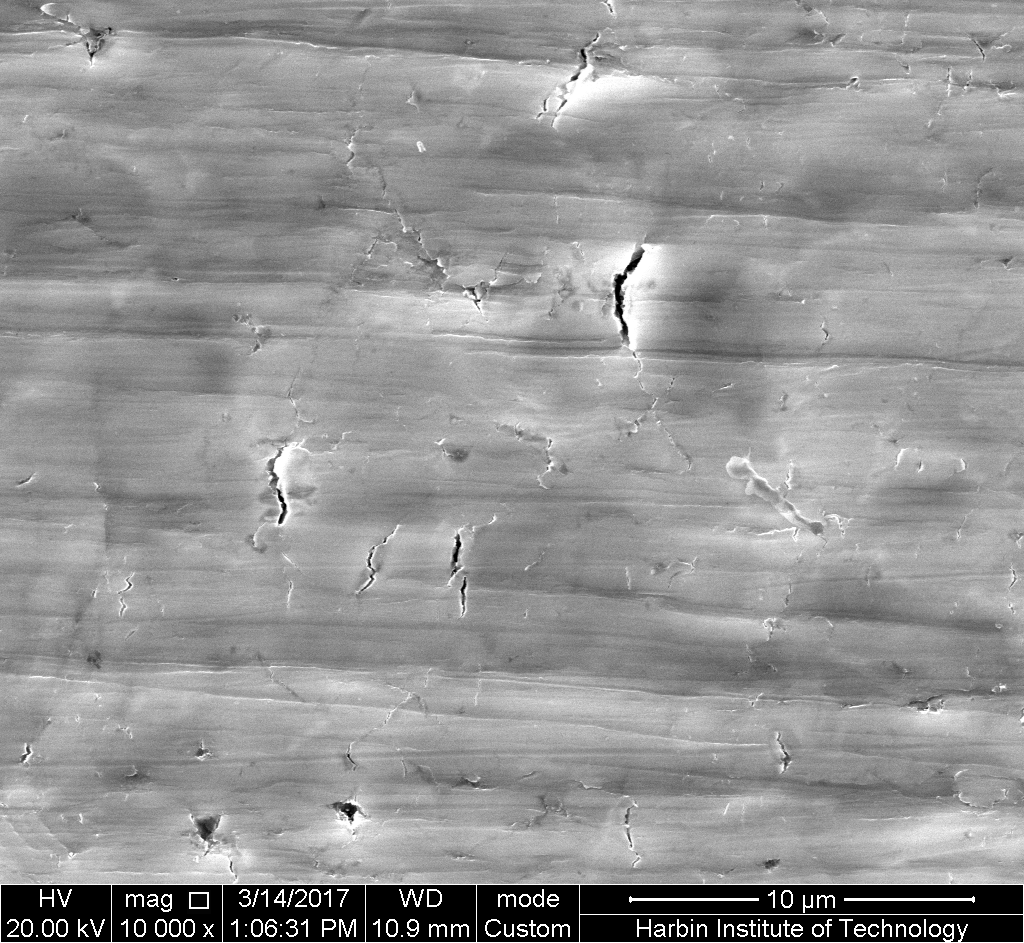


(b)


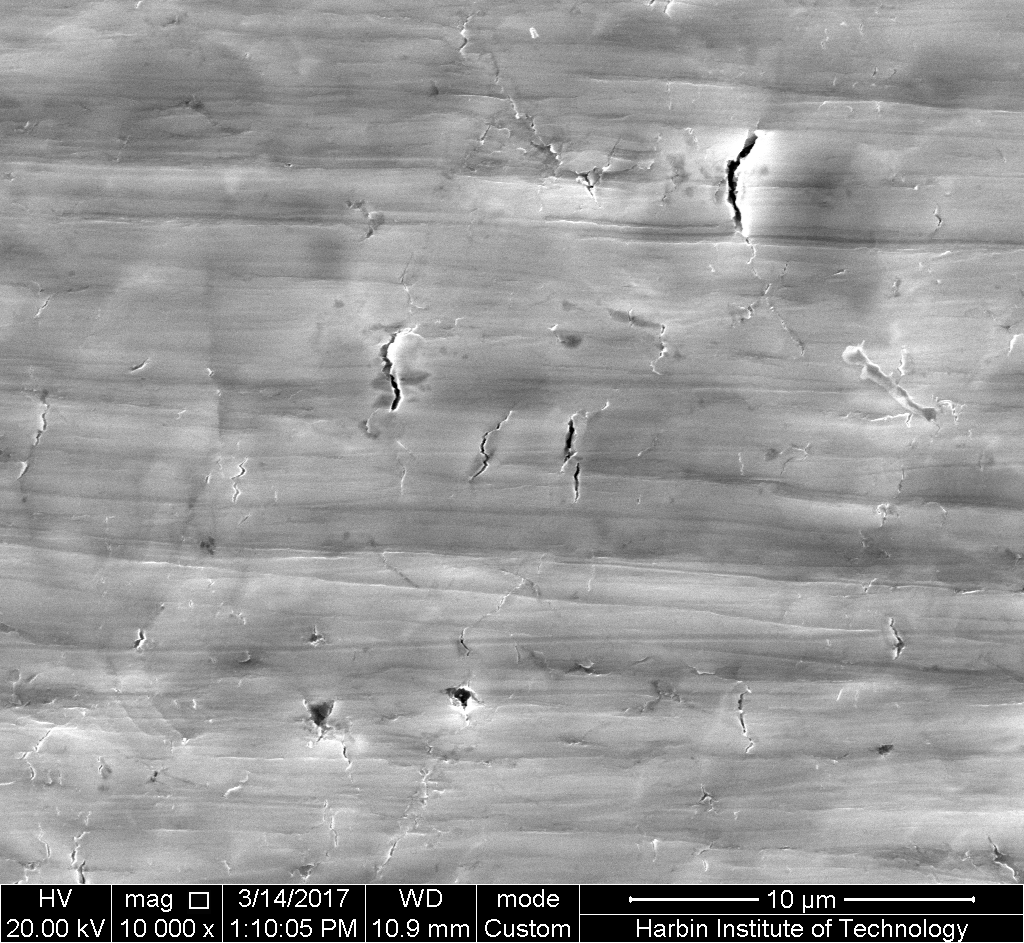


(c)


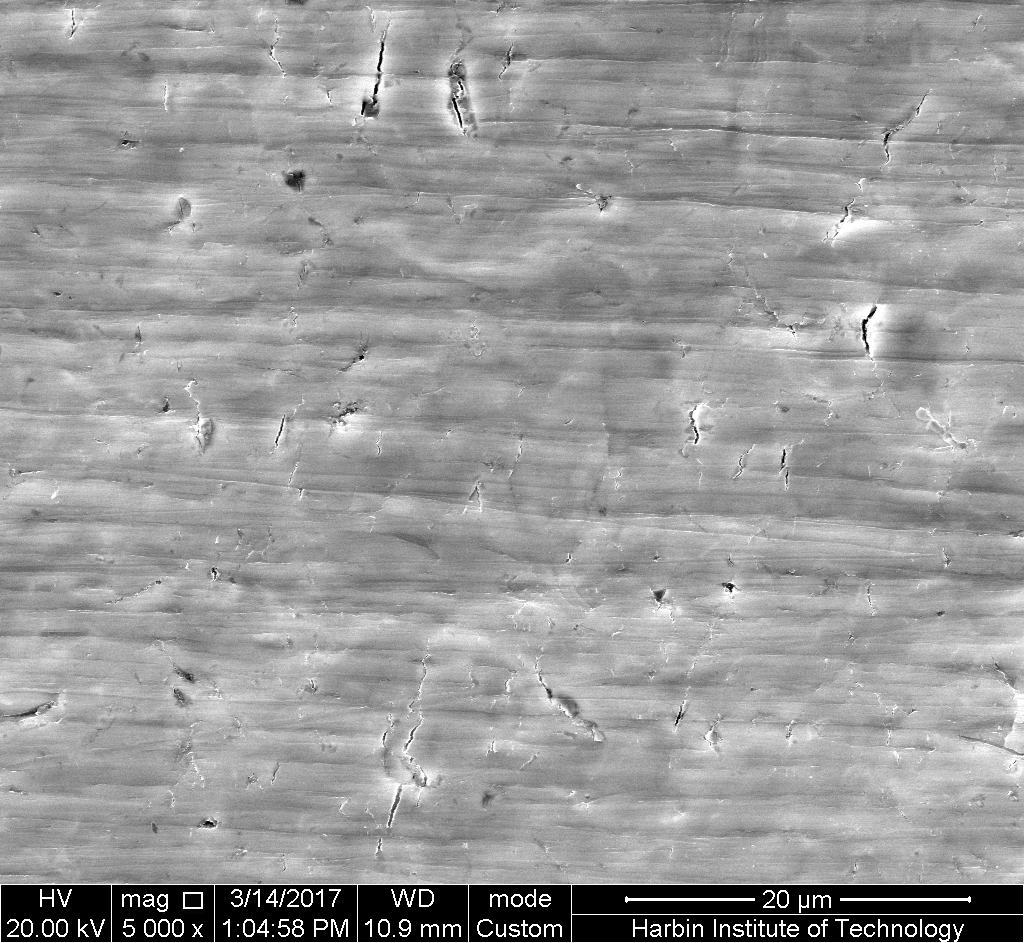


(d)


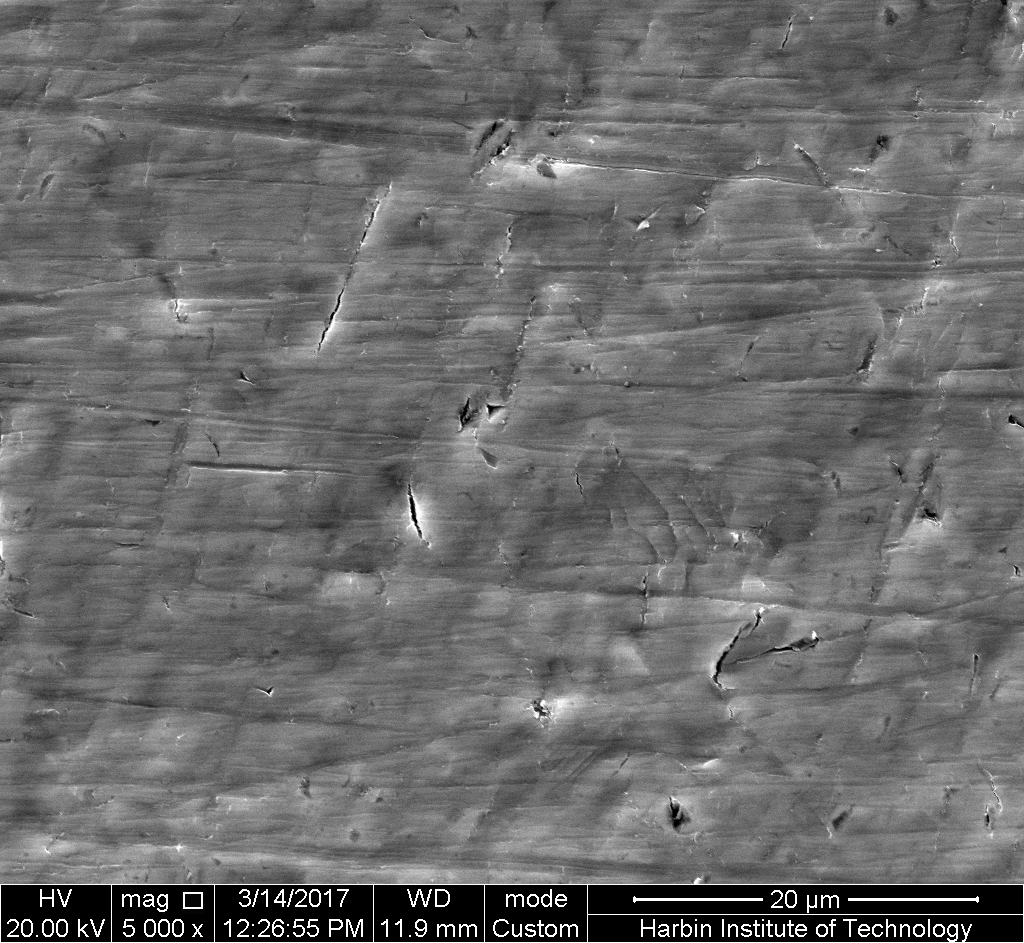


(e)


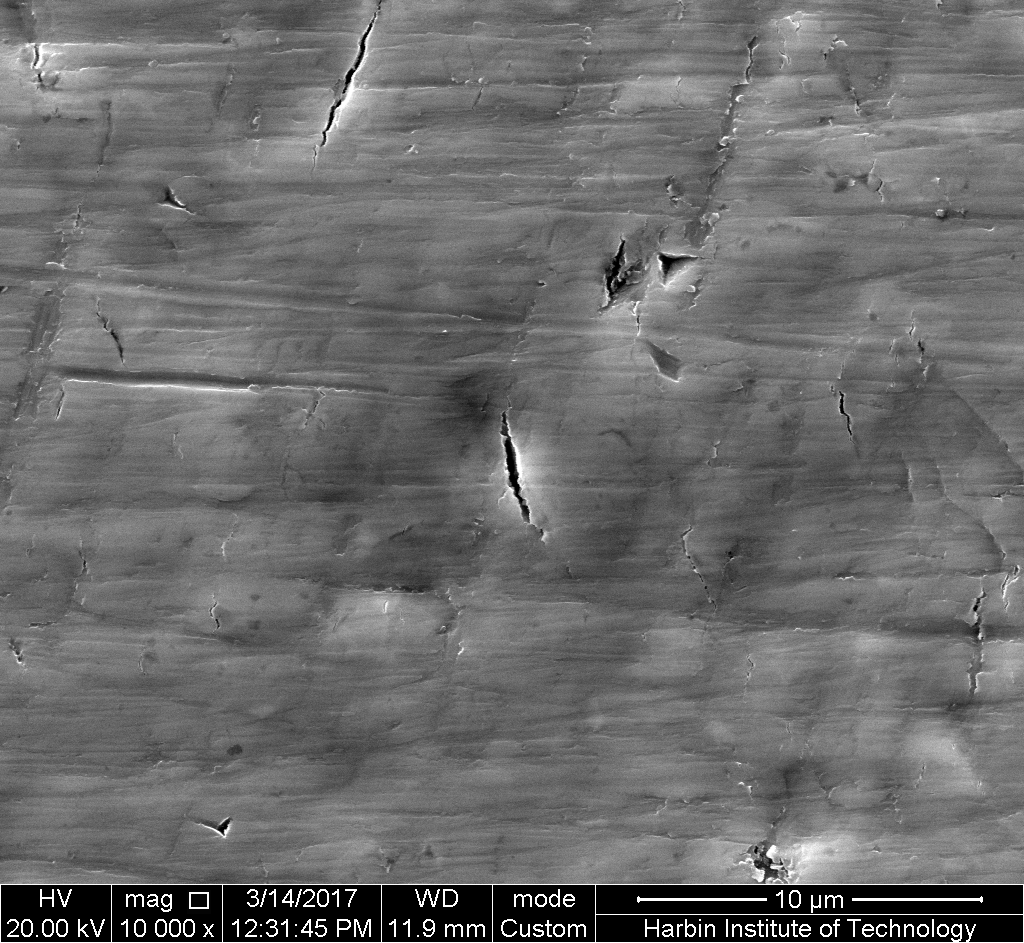


(f)

Supplementary Figure S15 Typical morphology of microcracks of damaged titanium alloy before electropulsing


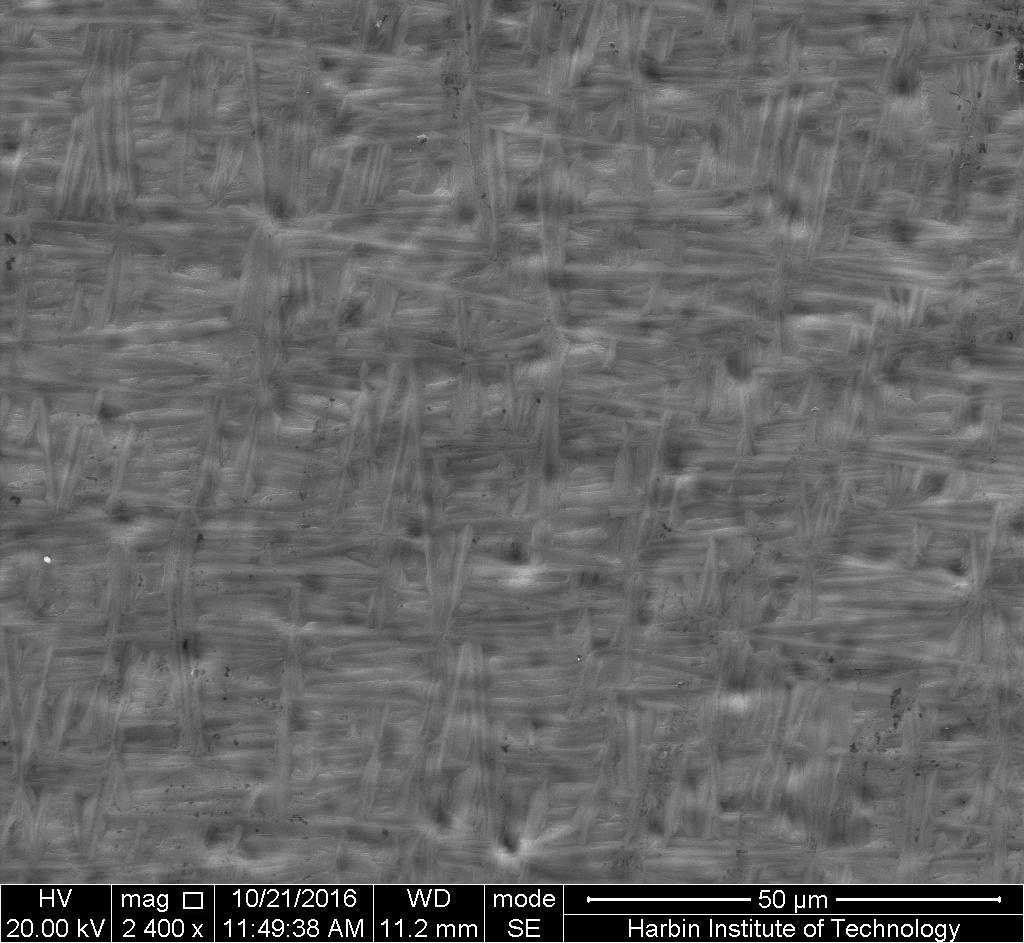


(a)


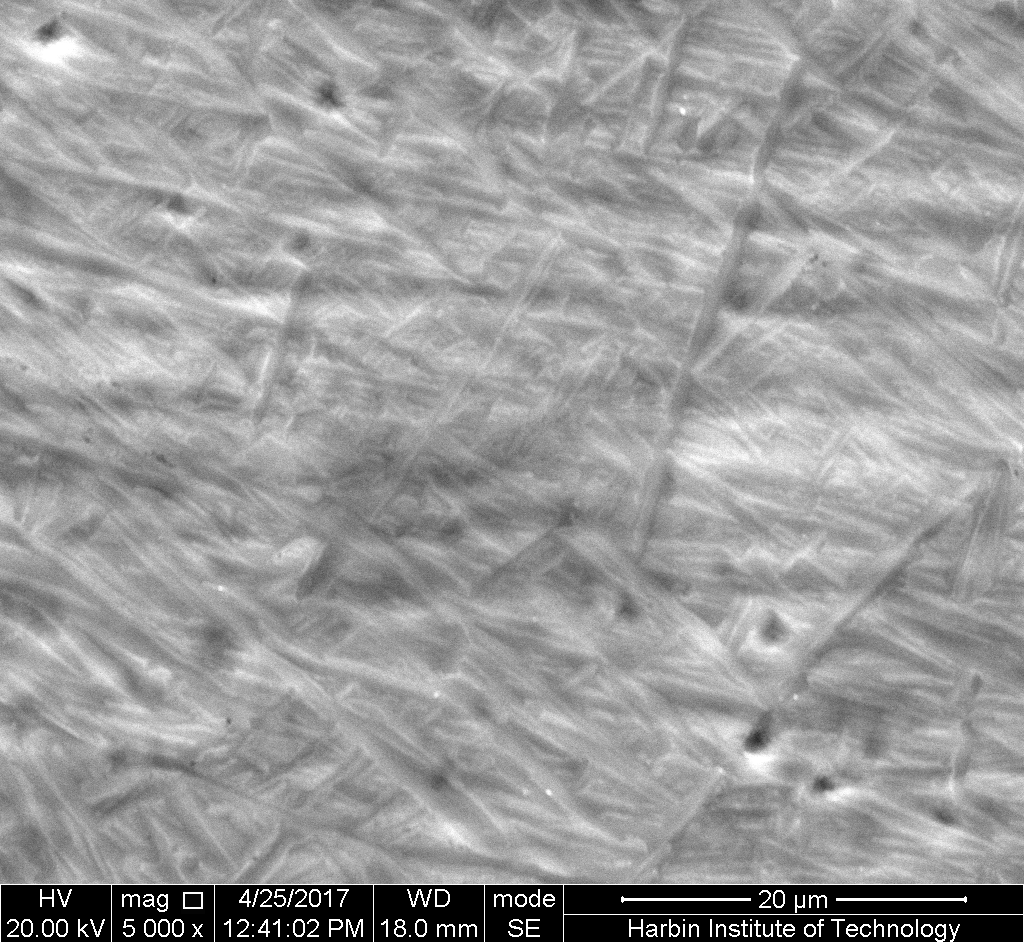


(b)


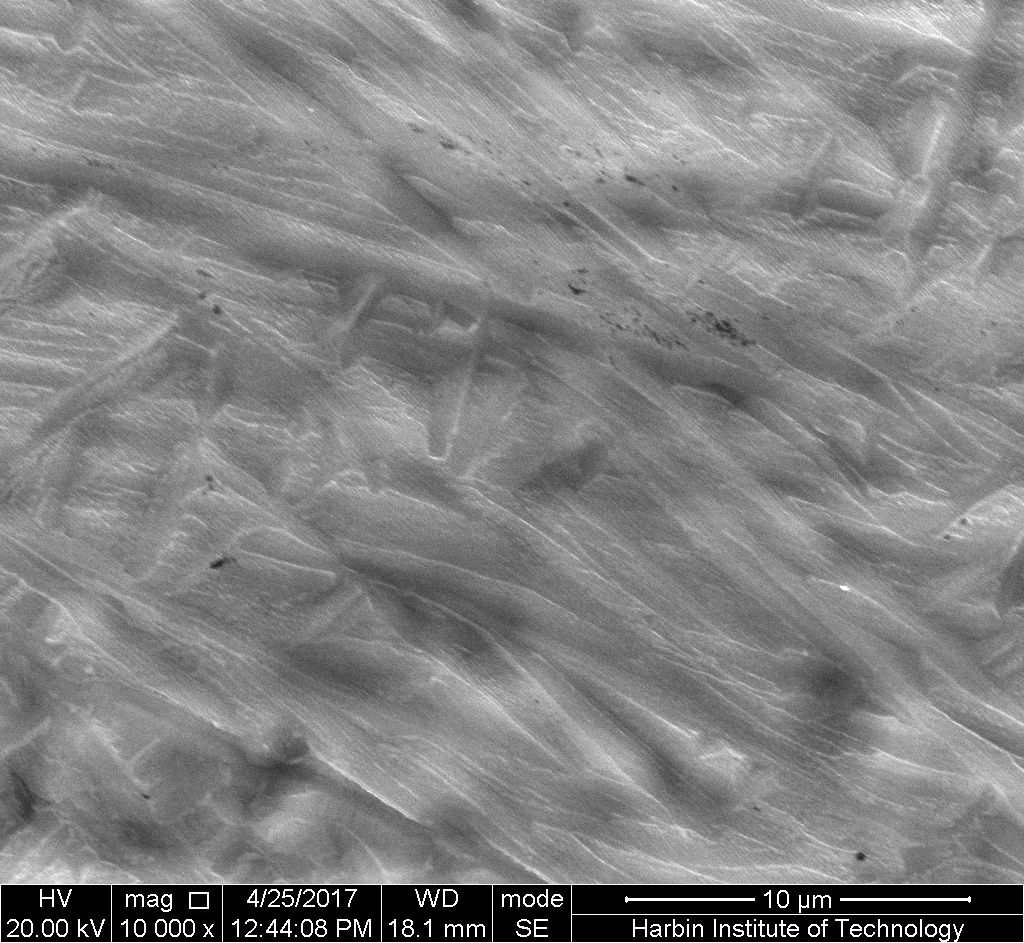


(c)

Supplementary Figure S16 Typical morphology of surface of damaged titanium alloy after electropulsing

**References**

1. Lecarme, L. et al. Heterogenous void growth revealed by in situ 3-D X-ray microtomography using automatic cavity tracking. Acta Mater. 63, 130–139. (2014)

2. Helbert, A.L., Feaugas, X., Clavel, M. Influence of stress triaxiality on the damage mechanisms in an equiaxed α/β Ti-6Al-4V alloy.  Metall. Mater. Trans. A. 27, 3043-3058(1996).

3.Helbert, A.L., Feaugas, X., Clavel, M. Effects of microstructural parameters and back stress on damage mechanisms in alpha/beta titanium alloys. Acta Mater. 46,939-951(1998).

4.Cheng, K. C. An analytical method of solution of the distribution function of crystal sizes in a volume. Acta Physica Sinica. 13,58-68(1957).

5. Bach, G. Stereology-Size distribution of particles derived from the size distribution of their sections (ed. Bach, G.). 174-186 (Springer, 1967)

6. Hilliard, J. E. Stereology-The direct determination of the number of convex particles per unit volume and the moments of their size distribution by an intercept analysis on a section (ed.Hilliard, J. E.). 195-196 (Springer, 1967)

7. Liu, T. J.C. Joule heating behaviors around through crack emanating from circular hole under electric load. Eng. Fract. Mech. 123,2–20(2014)
